# AGING AND SEXUAL RELATIONSHIPS: RECIPROCAL RELATIONS BETWEEN PHYSICAL PLEASURE AND EMOTIONAL SATISFACTION

**DOI:** 10.1093/geroni/igad104.3059

**Published:** 2023-12-21

**Authors:** Getrude Nyang’au, Pei-Shu Chao, Duane Rudy

**Affiliations:** University of Missouri - Columbia, Columbia, Missouri, United States; University of Missouri - Columbia, Columbia, Missouri, United States; University of Missouri - Columbia, Columbia, Missouri, United States

## Abstract

Previous research has found significant associations between physical pleasure and emotional satisfaction in sexual relationships. However, much of this research is cross-sectional and has focused on young and middle-aged adults. The present study examined data from the National Social Life, Health, and Aging Project (NSHAP). Physical pleasure and emotional satisfaction were assessed at three points in time (2005/2006; 2010/2011; and 2015/2016). Only data from individuals who were alive at all time points were used in the analysis. Three cohorts were examined: at time 1, the first cohort ranged in age from 57-64, the second cohort from 65-74, and the third cohort from 75-85. We examined reciprocal relations between physical pleasure and emotional satisfaction across time using a Random Intercepts Cross-Lagged Panel Model. The results revealed a positive reciprocal relationship between emotional satisfaction and physical pleasure among the first cohort, with emotional satisfaction at time 1 positively associated with physical pleasure at time 2, which in turn was positively associated with emotional satisfaction at time 3. For the second cohort, physical pleasure at time 2 predicted emotional satisfaction at time 3. Relationships among the two variables were not significant for the third cohort. The implication of this study would be to inform older adults in ongoing sexual relationships on the need to invest in activities that enhance physical pleasure and emotional satisfaction. Future research could examine what variables might account for reciprocal relationships being found for the first as opposed to the third cohort.

